# Gemella morbillorum endocarditis of pulmonary valve:a case report

**DOI:** 10.1186/s13019-017-0579-3

**Published:** 2017-03-23

**Authors:** Dan Li, Zhicheng Zhu, Xiaomei Zheng, Weitie Wang, Rihao Xu, Kexiang Liu

**Affiliations:** 1 Department of Cardiovascular Surgery, The Second Hospital of Jilin University, 218 Ziqiang Street, Nanguan District, Changchun, Jilin 130041 People’s Republic of China; 2 Department of Cardiovascular Surgery, The First Hospital of Tianjin, No. 186 Nankou Road, Tianjin, 300232 People’s Republic of China

**Keywords:** Infective endocarditis, Gemella morbillorum, Atrial septal defect, Ventricular septal defect, Double-chambered right ventricle

## Abstract

**Background:**

Pulmonary valve infective endocarditis is a rare finding for endocarditis. Infective endocarditis caused by Gemella morbillorum remains a scanty occurrence.

**Case presentation:**

This is a case reported of a 28-year-old Chinese male with endocarditis caused by pulmonary valve infection of *Gemella morbillorum* associated with congenital ventricular septal defect, atrial septal defect and double-chambered right ventricle. The patient presented with fever, shortness of breath, progressively worsening exertional fatigue, dyspnea and weight loss for 3 months. The diagnosis was made with transthoracic echocardiogram, blood cultures, and post-operative pathology. The patient developed congestive heart failure and was managed with aggressive antibiotic therapy followed by surgery. He underwent replacement of the pulmonary valve with an aortic bioprosthetic valve, repair of ventricular septal defect and atrial septal defect, reconstruction of the right ventricular outlflow tract, and excision of vegetations. His postoperative recovery was uneventful. No bacteria were isolated from the excised tissues. He was asymptomatic without recurrence at 3-month follow-up.

**Conclusions:**

The rare pathogen such as Gemella morbillorum can be the cause of infective endocarditis and timely surgical repair is necessary if the infection is refractory or there is progression of congestive heart failure under antibiotic cover.

## Background

*G. morbillorum* was originally described by Tunnicliff in 1917 [1]. The organism was relocated to the genus Gemella in 1988 [2]. Because of its characteristics of facultative anaerobic organism, G. morbillorum usually behaves as a commensal organism of the mucous membranes and is a part of the normal flora of human oropharynx, upper respiratory, gastrointestinal system, and genitourinary system [3]. It has been implicated as a causative pathogen in central nervous system infection, arthritis, pneumonia, pleural empyema, Ludwig’s angina, mediastinitis and osteomyelitis, hepatic abscess, peritonitis, and cardiovascular system infection.

Although *G. morbillorum* possesses very low virulence comparing to some other pathogens, it was reported to cause endocarditis [4–7]. The morbidity of *G. morbillorum* endocarditis remains a rare occurrence so far. Both native and prosthetic valves can be affected by *G. morbillorum*. Mitral and aortic valves were more commonly infected compared to tricuspid and pulmonary valve. Mitral and aortic valves are affected in almost equal number of cases, while the pulmonary valve is rarely involved. Pulmonary valve infective endocarditis (IE) caused by *G. morbillorum* is an extremely rare occurrence and only one case has been reported in the literature hitherto [8]. This is the 1st case reported of pulmonary valve IE caused by *G. morbillorum* in a young Chinese man who had a congenital ventricular septal defect (VSD), atrial septal defect (ASD) and double-chambered right ventricle (DCRV).

## Case presentation

A 28-year-old Chinese man was admitted to our hospital because of fever, chills and rigors, asthenia, malaise, cough, shortness of breath, edema and wasting for three days. His past medical history included an admission to the hospital 3 months ago due to bilateral pneumonia. At that time, the patient had intermittent, moderate grade fever, associated with chills and rigors. The fever was temporarily relieved with antipyretics. Laboratory studies showed white blood cells 12220/mm^3^ (82% neutrophils). Chest computed tomography demonstrated multiple patches high density in the upper right lung, multiple nodular high density in the lower right lung, a thick-walled cavity in the lower left lung, several swollen soft tissue shadows in the mediastinum, and an enlarged spleen. A transthoracic echocardiogram (TTE) showed VSD, ASD, DCRV with anomaly hypertrophied muscle bands in right ventricular outlflow tract (RVOT), continuous-wave Doppler study demonstrated a peak systolic gradient of 72 mmHg across the RVOT, trace tricuspid valve regurgitation, the thickened pulmonary valve and left ventricular ejection fraction (EF) of 57%. The diagnoses of pneumonia, congenital heart disease of VSD, ASD and DCRV were made. He was treated with cefminox sodium 2 g iv q12 h. He was afebrile in 72 h, and 14 days later he was discharged home in good condition. After discharge, the patient experienced intermittent, moderate fever and was treated with oral antibiotics. He had been complaining of edemas of lower extremities, wasting and 15-kg weight loss of 3 months’ duration.

On admission, the patient was febrile (38.3 °C), tachypnoic, anxious, and tachycardic with a heart rate of 110 bpm. His blood pressure was 120/80 mmHg. The patient was fully conscious with normal neurological examination. Auscultation of the chest was clear bilaterally without any adventitious sounds. There was a systolic murmur at the left sternal border and a diastolic murmur in the pulmonary valve area. An electrocardiogram revealed sinus tachycardia. Laboratory studies showed the following pathological values: hemoglobin 91 g/L, white blood cells 7100/mm^3^ (82% neutrophils), platelet count 88 000/mm3, albumin 28.9 g/L.

**Fig. 1:**
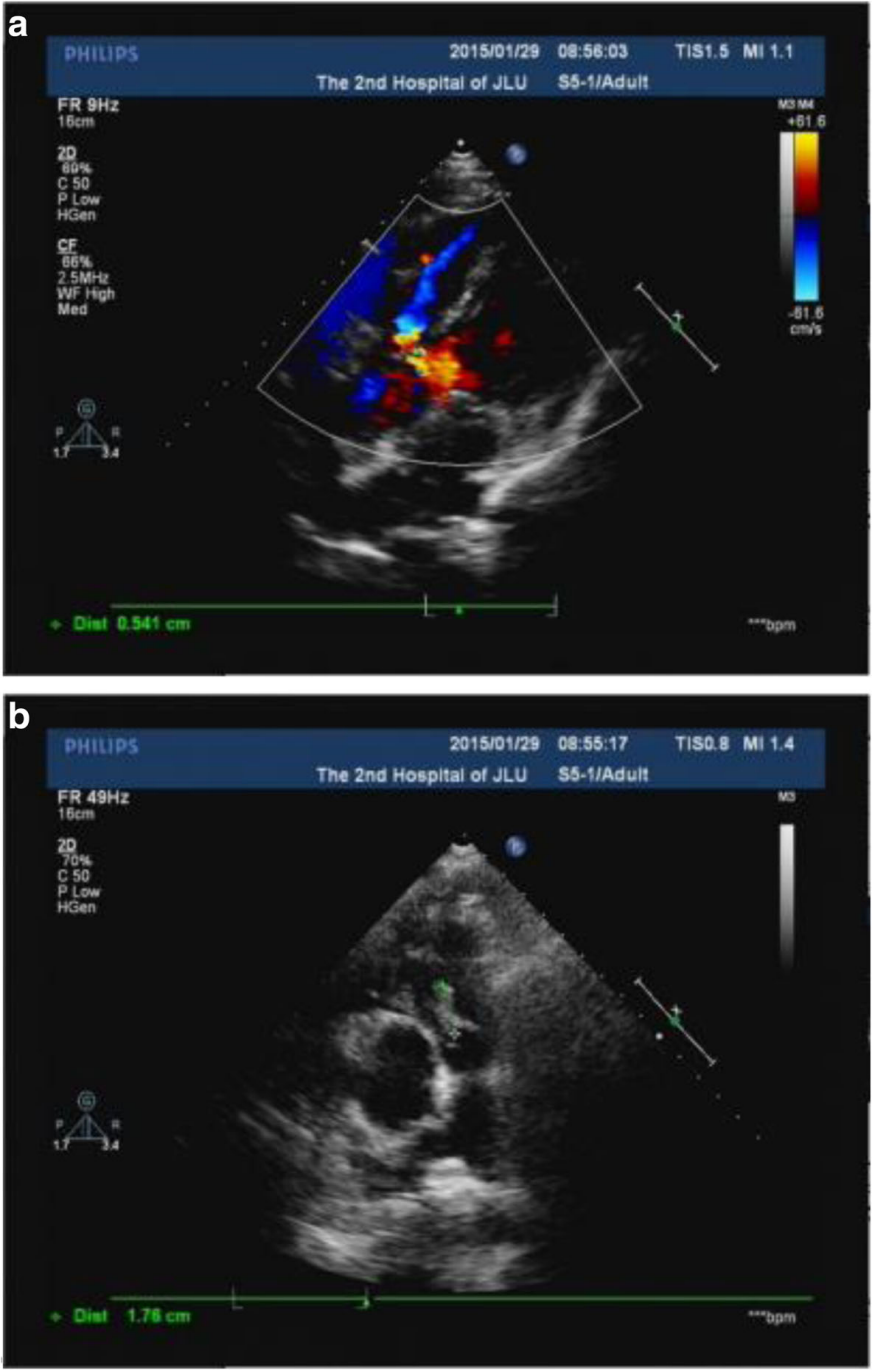
**a** Transthoracic parasternal short axis view showing vegetation (white arrow) on pulmonary valve. **b** Transthoracic echocardiography showing a small perimembranous type VSD with left to right shunt

A repeat TTE (Fig. 1) revealed a vegetation of 18 × 4 mm on the pulmonary valve with moderate pulmonary valve regurgitation, VSD, ASD and DCRV, and the other valves were unremarkable. Normal size of left ventricle was noted with an left ventricular EF of 54%. Three sets of blood cultures (each set contained one aerobic and one anaerobic vial) were obtained over a period of 24 h. Intravenous ceftriaxone and diuretics were initiated. After 2 days of treatment he became afebrile. On the third day of admission, all the three cultures grew alpha-hemolytic, catalase-negative Gram positive cocci. Susceptibility testing of the organism was done using the disc diffusion method. On the seventh day of admission, all of the three collected blood cultures (6 vials) grew *G. morbillorum*. The organism isolated was highly sensitive to penicillin, ceftriaxone, rifampicin, vancomycin and ciprofloxacin and therefore the above regimen was continued. On the eighth day of admission the patient’s clinical condition got worse with aggravated exertional fatigue and dyspnea, and TTE demonstrated severe pulmonary valve regurgitation. In light of these findings and the patient’s worsening clinical manifestations, a decision was made for urgent surgical treatment.

**Fig. 2:**
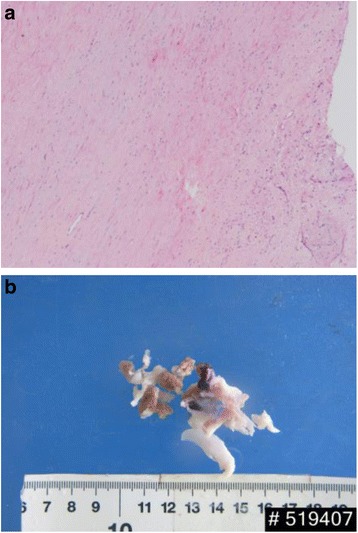
**a** The resected left and right valve leaflets of the pulmonary valves were severely destructed with a large vegetation on the anterior leaflet of the pulmonary valve. **b** Pathologic specimen of the valve tissue (pulmonary valve) showed fibrosis and myxoid degeneration

The surgical approach was via median sternotomy. After systemic heparinization, cardiopulmonary bypass (CPB) was instituted via ascending aortic and bicaval cannulation. After aortic cross-clamping, cardiac arrest was achieved through antegrade administration of cold blood cardioplegic solution. The perimembranous VSD (0.5 × 0.4 cm), ASD (0.5 × 0.6 cm) were repaired via an oblique right atriotomy. Tricuspid valve was normal, while left and right valve leaflets of the pulmonary valves were severely destructed with a large vegetation on the anterior leaflet of pulmonary valve observed. The leaflets of pulmonary valve and vegetation were excised (Fig. 2), and DCRV was corrected through pulmonary valve and tricuspid valve followed by fixation of aortic bioprosthetic valve into the pulmonic valve annulus. Anomalous septal and parietal muscle bundles seen in RVOT divided the right ventricle into a proximal high-pressure chamber and a distal low-pressure chamber, and they were carefully resected during surgery. The main pulmonary artery was then widened with a pericardial patch. The patient was rewarmed and successfully weaned from CPB.

The patient’s recovery was uneventful, while his temperature increased abruptly to 39.5 °C on the post-operative day 4. Laboratory studies showed white blood cells 20200/mm^3^ (89.7% neutrophils). Blood cultures with three separate blood specimens were obtained. In addition to previous medications, vancomycin 2 × 1 g/day IV was added. He became afebrile in 2 days with the combination of antibiotics. Vancomycin was discontinued in 1 weeks, while ceftrixone was continued. Subsequent blood cultures showed negative. Culture of the excised tissue specimen was also negative for bacteria. The patient received 4 weeks antibiotic treatment and recovered well after surgery. He was discharged in good condition and remained asymptomatic at his 3-month follow-up.

## Discussion

*G. morbillorum* was originally isolated by Tunnicliff in 1917. Infections caused by *G. morbillorum* are rare, and *G. morbillorum* endocarditis is a rare clinical entity. About 45 cases of *G. morbillorum* endocarditis were reported in the literature hitherto.

Predisposing factors for *G. morbillorum* endocarditis include underlying cardiac pathologies, such as pre-existing valvular lesions and congenital heart diseases, etc. Any of the valves can be affected, including native valve and prosthetic valve. It has been reported that mitral and aortic valves were affected in an almost equal number of cases, sometimes even simultaneously; while the tricuspid valve and pulmonary valve is only rarely involved.

Our patient met the Duke’s criteria [9] for IE. The major criteria met included positive echocardiographic evidence of vegetations, worsening pulmonary valvular regurgitation, and positive blood culture. The minor criteria met by this patient included fever, the predisposing heart condition of a congenital VSD, ASD and DCRV. The definitive source of infection for the organism was not found. Pulmonary valve IE is a rare finding accounting for less than 2% of hospital admissions for endocarditis [10]. The majority of pulmonary valve IE cases (55%) occurred in patients with congenital heart disease. The occurrence of pulmonary valve IE caused by *G. morbillorum* is extremely rare. Only one case of pulmonary valve IE caused by *G. morbillorum* in a case of ASD was reported in the literature so far [8]. ASD can be a cause of the pulmonary valve endocarditis which is subjected to damage due to increased blood flow although it is not included in the list of predisposing factors for *G. morbillorum* endocarditis. VSD is a risk factor of IE involving right heart structures. Birkenkamp et al. speculated that the high pressure flow created by the left-to-right shunt across the VSD damaged the pulmonary valve, making the valve more susceptible to bacterial infection [11]. Our patient suffered from pulmonary valve IE caused by *G. morbillorum* with ASD, VSD and DCRV. Blood cultures were taken when he was admitted to our hospital. After specimens of blood for culture were drawn, he was treated with susceptible antibiotics. After 2 days of treatment he became afebrile. But on the eighth day of admission the patient’s conditions deteriorated with worsening pulmonary valve regurgitation and secondary heart failure which were difficult to be controlled by the medical treatment, we referred him for urgent surgical intervention to excise vegetations and infected tissue thoroughly, correct all cardiac defects and replace the pulmonary valve. The patient continued his antibiotics therapy after operation to control the recurrent of infection. But his temperature increased abruptly and the count of white blood cells also increased on the post-operative day 4, another sensitive antibiotics vancomycin was added. He became afebrile with the combination of antibiotics. To prevent the recurrence of the infection, the patient received 4 weeks of antibiotics coverage. He recovered well and was discharged from the hospital.

## Conclusions

The patients of *G. morbillorum* endocarditis of pulmonary valve can be treated effectively with sensitive antibiotics. While the patient presents abrupt worsening and refractory heart failure, one of the key factors for the patient to recovery is urgent surgical repair of all cardiac defects, excision of vegetations and infected tissue.

## Abbreviations

ASD: Atrial septal defect
CPB: Cardiopulmonary bypass
DCRV: Double-chambered right ventricle
EF: Ejection fraction
G. morbillorum: Gemella morbillorum
IE: Infective endocarditis
RVOT: Right ventricular outlflow tract
TTE: Transthoracic echocardiogram
VSD: Ventricular septal defect
